# Prevalence and Outcome of Operative Vaginal Delivery among Mothers Who Gave Birth at Jimma University Medical Center, Southwest Ethiopia

**DOI:** 10.1155/2018/7423475

**Published:** 2018-07-09

**Authors:** Zenebe Hubena, Ahadu Workneh, Yibeltal Siraneh

**Affiliations:** ^1^Kuyu General Hospital, North Showa, Oromia Regional State, Ethiopia; ^2^Department of Obstetrics and Gynecology, Faculty of Medical Sciences, Institute of Health, Jimma University, Ethiopia; ^3^Department of Health Economics, Management and Policy, Faculty of Public Health, Institute of Health, Jimma University, Ethiopia

## Abstract

**Background:**

Operative vaginal deliveries (OVD) are vaginal deliveries accomplished with the use of a vacuum device or forceps. If it is technically feasible and can be safely accomplished, termination of second stage labor by operative vaginal delivery is indicated in any condition threatening the mother or fetus that is likely to be relieved by delivery. Hence, the objective of this study is to assess the prevalence, common indication, outcome, and associated factors of operative vaginal delivery among mothers who gave birth in Jimma University Medical Center (JUMC).

**Method:**

A facility-based cross-sectional study design was used in maternity ward on 242 mothers who gave birth by operative vaginal delivery from December 01, 2016, to May 30, 2017. The clinical data were collected using a check list, recordings of intrapartum fetal and maternal state, and immediate fetomaternal outcomes. The study participants were recruited using consecutive sampling method. Sociodemographics and related data were collected at exit using structured interviewer administered questionnaire which was developed by reviewing different literature and the remaining information abstracted from patient charts. Data were entered to Epidata 3.1 and exported to SPSS version 21 for analysis. Bivariate analysis was done to identify candidate variables using p<0.25. Multivariable logistic regression was used to control the effect of confounding variables and to identify factors affecting the fetomaternal outcome. Statistical significance was declared at P<0.05 using adjusted OR with 95% CI.

**Result:**

Out of the 2348 pregnant mothers who gave birth in the labor ward of JUMC during the 6 months of the study period, 242 (10.3%) were by operative vaginal delivery (OVD). The commonest indication for operative vaginal delivery is found to be nonreassuring fetal heart rate pattern, 136 (56.2%). Out of all neonates delivered by operative vaginal delivery 210 (86.8%) had favorable outcome. Of all mothers who gave birth by operative vaginal delivery 232 (95.9%) had favorable outcome. Type of instrument used for operative vaginal delivery (AOR=0.228, 95%CI: 0.078, 0.671) and presence of grade two (AOR=0.163, 95%CI: 0.031, 0.858) and grade three (AOR=0.088,95%CI: 0.024,0.327) meconium stained amniotic fluid are factors affecting neonatal outcome while neonatal birth weight (AOR=0.007, 95%CI: 0.000, 0.151) is factor affecting maternal outcome of operative vaginal delivery.

**Conclusion:**

Prevalence of operative vaginal delivery is found to be 10.3% with the commonest indication of nonreassuring fetal heart rate pattern. Nearly all of mothers and neonates had favorable outcome. Type of instrument applied for operative vaginal delivery is the strongest predictor of neonatal outcome while neonatal birth weight is the only predictor of maternal outcome identified in this study.

## 1. Background

Operative vaginal deliveries are vaginal deliveries accomplished with the use of a vacuum device or forceps. If it is technically feasible, it can be safely accomplished. Termination of second stage of labor by operative vaginal delivery is indicated in any condition threatening the mother or fetus. According to the birth certificate data from the National Vital Statistics Report, forceps or vacuum-assisted vaginal delivery was used for 3.6% of births in the United States in 2010, and it accounts for around 11% and 17.3% of births in Royal College of obstetricians and gynecologists, Australia, and in Tikur Anbessa Specialized hospital, Ethiopia, respectively [1–4].

When prerequisites have been met, the appropriate indications for consideration of either forceps delivery or vacuum extraction are prolonged second stage, nonreassuring fetal heart rate tracing, or shortening of the second stage of labor for maternal benefit. Both forceps and vacuum have the potential to cause fetal and neonatal injury; however, the incidence of maternal injury is less with the vacuum than with forceps. In order to minimize both maternal and fetal risks, the operator must be familiar with the indications, contraindications, application, and use of the particular instrument. It is recommended that OVD should be performed from either a low or outlet station [5]. Studies revealed that prevalence of OVD ranged from 3 to 11% in different settings [2, 6].

Vacuum extractor is less likely to achieve a successful vaginal delivery and to cause serious maternal injury than applying the forceps. Although the vacuum is associated with a greater incidence of cephalohematoma, other facial/cranial injuries are more common with forceps [7].

Although operative vaginal delivery may be performed, as infrequently as in 1.5% of deliveries in some countries, it may be as high as 15% in other countries. In the United Kingdom, the rates of instrumental vaginal delivery range between 10% and 15%; these rates have remained fairly constant, although there has been a change in preference of instrument [6].

But currently studies show that there is a decreasing trend of instrumental deliveries and is a major concern in health care system all over the world. Assessing the trends of instrumental deliveries and its major indications would be useful in adopting suitable measures to reduce the caesarean section rate and the problems associated with it. A five-year retrospective study conducted on trends of instrumental deliveries at a tertiary teaching hospital in Puducherry, India, showed among a total of 5445 deliveries that occurred during study period, 7.7% were instrumental vaginal deliveries. The year-wise rate of instrumental deliveries ranges from 6.1% to 9.8%. During the study period (except during year 2011), a declining trend for instrumental deliveries was observed [8].

Studies revealed that the most common indication for OVD is to shorten second stage of labor considering maternal condition and the commonest unfavorable outcomes of OVD varies. Study done in Shankar Nagar and Raipur, India, reported that the most common indication was to cut short second stage of labor (52.5%) (preeclampsia, heart disease) followed by prolonged second stage of labor (22.5%), fetal distress, and maternal exhaustion. The risk of neonatal morbidity was similar between infants delivered by vacuum or forceps [9]. The commonest maternal complication was postpartum hemorrhage and genital tract laceration [10]. Evidence evaluating neonatal morbidity after instrumental vaginal delivery is inconsistent. A systematic review of 10 trials comparing vacuum extraction with forceps delivery found no significant differences in APGAR scores at one and five minutes and few serious injuries in neonates, although the vacuum extractor was associated with an increase in cephalhematoma and retinal hemorrhage [11]. In JUMC, no study conducted to assess the prevalence, indications, fetomaternal outcome, and associated factors of operative vaginal delivery.

## 2. Methods and Participants

### 2.1. Study Area and Period

The study was conducted in Jimma University Medical Center (JUMC) which is located 352kms Southwest of Addis Ababa. JUMC is found in Jimma zone of Oromia regional state within Jimma Town. It is one of the oldest specialized teaching hospitals (currently renamed as medical center) in the country giving services to people living in Jimma zone and serve as a referral hospital in the Southwest Ethiopia. It is teaching center for many clinical undergraduate and postgraduate specialty students. Department of Obstetrics and Gynecology has two inpatient (gynecology and obstetrics), one maternal health clinic, one gynecologic OPD, one family planning clinic, and referral clinics (gynecology oncology, benign gynecologic diseases, and high risk pregnancy). It has eight obstetricians and gynecologists and 32 residents from year I to III. The labor ward has 7 beds in first stage and 4 delivery couches. Maternity ward has 50 beds, 2 emergency operation rooms, and one recovery room with 2 beds and 2 resuscitation tables for newborns. There were a total of 2,654 deliveries recorded over eight months from November 1, 2015, to June 30, 2016, of which 266 were by OVD.

### 2.2. Study Design and Population

A facility-based cross-sectional study design was used. All mothers who gave birth at JUMC during the study period were the source population. All mothers who gave birth by operative vaginal delivery were included. All mothers for whom OVD indicated and fulfilled prerequisites (fetal head being engaged, vertex presentation, cervix being fully dilated (8cm for ventouse), membranes ruptured, exact position of head known, fetal size estimated (weight and ga), informed consent, maternal bladder being empty, adequate maternal pelvis, and back-up plan in place in case of failure to deliver) were eligible. Mothers for whom OVD was indicated but with IUFD and fetus with congenital anomaly were not eligible.

### 2.3. Sample Size Determination

The sample size was determined using a single population proportion formula n= z^2^p (1-p)/d,^2^ where p (17.3%) is the estimate of the proportion of operative vaginal delivery elsewhere. Considering all recommended values for each parameter, the sample was estimated to be 220. By adding 10% of this sample size for expected nonresponse rate, the final sample size becomes 242. Prevalence of OVD was taken 17.3% as obtained from a retrospective study conducted in 2004 at Tikur Anbessa Specialized Hospital, Addis Ababa, Ethiopia. All mothers who gave birth by OVD during study period were included using consecutive sampling technique till the required sample size completed.

Study variables were fetal parameters (FHR, GA, neonatal birth weight), obstetric related variables (cervical dilatation, uterine contraction/maternal effort, descent/station, need for rotation, indication for OVD, type of instrument used for OVD, timing of application of OVD (on arrival/followed), status of liquor), maternal parameters (age, parity, residency, ANC follow-up), fetomaternal outcomes (serious maternal morbidity or death, postpartum hemorrhage, blood transfusions, episiotomy extension, third and fourth degree tears, cervical laceration, need for major surgery (hysterectomy, urinary retention and bladder dysfunction, low APGAR score, admission to NICU, need for resuscitation at delivery, neonatal sepsis at neonatology, birth trauma (fractured bone, cephalhematoma), and condition at discharge (normal, improved, died).

### 2.4. Data Collection Tools and Procedures

The tool was developed by reviewing different literature and translated into local languages (Afan Oromo, Amharic) and then back translated to English by third party to check its consistency. Structured interviewer administered questionnaire used to interview mothers at exit. Two obstetrics and gynecology residents and 3 midwives were trained on how to interview eligible mothers and abstract information from respective charts. Checklist was used to extract data from the patient chart. The first part required information about patient's age, gravidity, parity and estimated gestation age. Second part required the parameters of labor which were fetal heart rate, liquor state, cervical dilatation, descent of head, uterine contraction, and maternal BP. The third part required the fetal outcomes which were assessed in terms of live birth (APGAR score at first and fifth minutes), need of resuscitation, admission to neonatal ward for special care, and the reasons for admission. The fourth part included information about the mode of delivery (OVD) and immediate maternal outcomes. Immediate maternal outcomes were recorded as favorable and unfavorable if the woman got PPH, perineal tear (third degree and above), need of blood transfusion, urinary bladder injury, hysterectomy, or bowel injury.

### 2.5. Operational Definitions

#### 2.5.1. Operative Vaginal Deliveries (OVD)

Vaginal deliveries are accomplished with the use of a vacuum device or forceps.

#### 2.5.2. Indication of OVD

It means any condition threatening the mother or fetus that is likely to be relieved by immediate delivery when prerequisites are fulfilled.

#### 2.5.3. Asphyxia

Asphyxia is a condition in which viable newborn fails to attain or initiate respirations after delivery.

#### 2.5.4. APGAR Score

APGAR score is method of assessing fetal conditions at time of delivery.

#### 2.5.5. Low APGAR Score

APGAR score of less than seven is considered low.

#### 2.5.6. Birth Trauma

Birth trauma is any trauma to the newborn as a result of labor and delivery like cephalohematoma, subgaleal hemorrhage, retinal hemorrhage, shoulder dystocia, clavicular fracture, and scalp lacerations.

#### 2.5.7. Favorable Outcome

If mother and neonate has no complications, this is considered a favorable outcome.

#### 2.5.8. Unfavorable Outcome

The unfavorable outcome is when mother and neonate developed complications (maternal complications like PPH, genital tear, need of blood transfusion, need of major surgery, death, and neonatal complications like low APGAR score, need of resuscitation, admission to NICU, and neonatal death).

#### 2.5.9. Episiotomy Extension

Episiotomy extension is an incision that is deeper or longer than is necessary to permit the birth of newborn.

### 2.6. Data Processing and Analysis

The collected data were cleaned, entered in to Epi-data 3.1, and exported to SPSS for windows version 21 for data analysis. Descriptive statistics used to describe the main features of the data. Bivariate analysis was done to identify candidate variable using p<0.25. Multivariate Logistic regression was used to control the effect of confounding variables. Variables having P<0.25 from bivariate analysis were included in multivariable logistic regression analysis. Finally, statistical significance declared at P<0.05 using adjusted OR with 95% CI.

### 2.7. Ethical Considerations

Ethical clearance was taken from Institutional Review Board (IRB) of Jimma University Institute of health, and permission letter was obtained from JUMC including Obstetrics and Gynecology Department. Participants were informed about the objective of the study and relevant issues before informed consent taken. Confidentiality was assured by using codes and their privacy was also kept.

## 3. Results

### 3.1. Sociodemographic Characteristics

Response rate for this study is 100%. Out of 2348 laboring mothers who gave birth in the labor ward of JUMC during the 6 months of the study period 242(10.3%) were by operative vaginal delivery(OVD). Out of all 92 (38%) of them were in the age group of 20-24 years, the mean age of study participants was 24.7years +/-5years SD. Most 144(59.5%) of them were from outside of Jimma Town. Almost all 237 (97.9%) of them were married, and majority of them were Muslim by religion, Oromo by ethnicity, and housewives by occupational status, 161(66.5%), 201(83.1%), and 101(41.7%) respectively. Those mothers who cannot read and write and with educational level of grade 1-8 each account for one-third of cases and 128(52.9%) of mothers' monthly income is 500-1742ETB. (See Table 1)

### 3.2. Obstetric Related Variables

**Figure 1:**
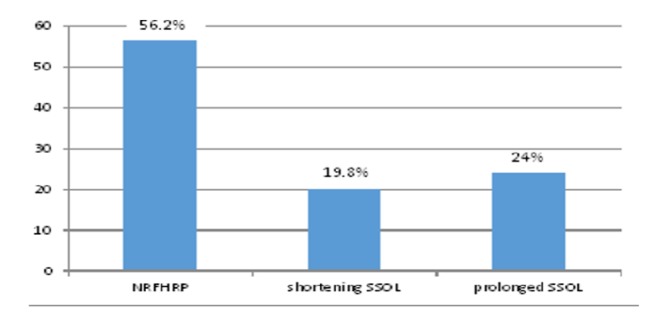
Proportion of indications for OVD among mothers who gave birth by OVD in JUMC December 1, 2016, to May 30, 2017.

According to this study 168(69.4%) of mothers were primiparas and 233(96.3%) of mothers who gave birth by OVD had at least one ANC visit and 118(48.8%) had four or more ANC visits. The commonest indication for OVD is found to be NRFHRP 136(56.2%) which is followed by prolonged SSOL 58(24.0%). Of the types of OVDs forceps is more commonly used 192(79.3%) and vacuum deliveries were 50(20.7%) with ratio of 4:1. Most 132(54.5%) of the applied classification of OVD is low forceps or low vacuum and 110(45.5%) were outlet forceps or outlet vacuum. Out of 173(71.5%) OVDs applied for those mothers who were being followed at labor ward of JUMC, 70 (28.9%) of the laboring mothers had different grades of MSAF. The GA at delivery was between 37 and 42 weeks in 213 (88.0%) of the laboring mothers. Fifteen mothers (6.2%) had postterm pregnancy and 14(5.8%) were preterm deliveries. Majority 221(91.3%) of the newborns weigh 2500-3999 grams. (See Table 2 and Figure 1)

### 3.3. Neonatal Outcome and Maternal Sociodemographics

Of all mothers who gave birth by OVD during the study period 210(86.8%) had favorable neonatal outcome. Mothers in the group of 25-29 years and those from Jimma Town had the higher proportion of favorable neonatal outcome which is 71(89.9%) and 90(91.8%), respectively. Neonates of mothers who are farmers in occupation had the lowest proportion 64(78%) of favorable outcome and the proportion of those neonates from mothers who at least can read and write that had favorable outcome is higher 149(90.3%) when compared with proportion among illiterates 61(79.2%). Mothers whose monthly income is greater than 1743ETB had higher 98(94.2%) proportion of favorable neonatal outcome and 205(86.5%) of those mothers who are married had neonates with favorable outcome. (see Table 3)

### 3.4. Neonatal Outcome and Obstetric Related Variables

The proportion of neonates who had favorable outcome among para II-IV mothers and neonates born at term is higher which is 96.2% and 87.3% respectively. Those mothers who had at least one ANC visit have higher proportion of favorable neonatal outcome (88.0% versus 55.6%) and those who had four and above visits have better proportion 91.5% with favorable outcome. Based on indication NRFHRP has the lower proportion of favorable outcome. Forceps deliveries are having higher proportion of favorable outcome (91.7% versus 68%) and the same holds for outlet (90.9%). The higher proportion of mothers who were followed at the labor ward of JUMC had favorable neonatal outcome than those for whom OVD is applied on arrival (91.7% versus 75.4%). Half of those with G3MSAF have favorable outcome (see Table 4).

Of the 242 OVDs, neonates with low APGAR score (4-6) at first and fifth minutes were 95(39.3%) and 16(6.6%), respectively, and those with very low APGAR score (0-3) at first and fifth minutes were 10(4.1%) and 2(0.8%), respectively, and 47(19.4%) needed resuscitation. There were a total of 34(14.0%) admissions to NICU and 3 neonatal deaths before referral to NICU. Majority of the admissions were for MAS 15(44.1%) followed by MAS+ subgaleal hemorrhage 10(29.4%). There were different birth injuries like SGH, skull fracture, and bruising with a total of 15(44.1%) injuries (one neonate with SGH requiring blood transfusion). The proportion of neonates with birth injury is higher among vacuum group than forceps deliveries (20% versus 2.6%). Of the admitted cases to NICU 26(76.5%) were improved, 6(17.6%) died, and status of two of the cases is unknown. The commonly ascribed causes of neonatal deaths were respiratory failure, PNA, and multiorgan failure. (See Table 4)

### 3.5. Maternal Outcome and Sociodemographic Variables

Out of 242 mothers, 232(95.9%) have favorable maternal outcome. Mothers in the group of 20-24 years had the higher proportion of favorable maternal outcome which is 97.8% and all mothers from Jimma Town have favorable maternal outcome. Those mothers who earn >1742ETB per month have higher proportion 102(98.1%) of favorable maternal outcome. (See Table 5)

### 3.6. Maternal Outcome and Obstetric Related Variables

The proportion of mothers who had favorable outcome among para II-IV mothers and for whom prolonged SSOL is an indication for OVD is higher which is 98.1% and 98.3%, respectively. Based on type of OVD used the proportion of mothers with favorable maternal outcome is almost equal between forceps and vacuum deliveries (95.8% versus 96.0%). Almost all (99.1%) of outlet forceps/vacuum deliveries have favorable maternal outcome. Of all 242 OVDs 8 cases (3.3%) were complicated with PPH and the PPHs were secondary to three uterine atony and five episiotomy extension cases. Two of the eight PPH cases underwent per partum hysterectomy and three of them were transfused. There is one case complicated with fourth degree genital tear with no PPH and one maternal death after forceps is applied for shortening of SSOL for cardiac illness and the death is ascribed to be secondary to cardiac arrest. (See Table 6)

### 3.7. Factors Affecting Neonatal Outcome

Place of residence, occupation, income, parity, indication for OVD, type of instrument used for OVD, station at which OVD is applied, time of application, and status of liquor were variables identified as a candidate variable from bivariate logistic regression analysis and then fitted into the final multivariable logistic regression model using enter method to identify independent factors affecting the fetal outcome. This study shows that there is significant association between type of instrument used for OVD and neonatal outcome, 80% of mothers who gave birth by vacuum are less likely to have favorable neonatal outcome than those with forceps deliveries (AOR=0.228, 95%CI: 0.078, 0.671). Meconium stained amniotic fluid had shown association with neonatal outcome, 84% of mothers with G2MSAF (AOR=0.163, 95%CI: 0.031, 0.858) and 90% of mothers with grade 3 MSAF (AOR=0.088,95%CI: 0.024,0.327) are less likely to have favorable neonatal outcome than those with clear amniotic fluid. (see Table 7)

### 3.8. Factors Affecting Maternal Outcome

Income, indication for OVD, station at OVD application, and weight of newborn at delivery were variables identified as candidate variable from bivariate logistic regression analysis and then fitted into the final multivariable logistic regression model using enter method to identify independent factors affecting the maternal outcome. Neonatal birth weight had shown strong association with maternal outcome, almost all mothers with neonatal birth weight >4000grams (99.6%) are less likely to have favorable maternal outcome than those with neonatal birth weight of 2500-3999grams (AOR=0.007, 95%CI: 0.000, 0.151), respectively. (see Table 8)

## 4. Discussion

Prevalence of OVD application is 10.3% in JUMC and the finding is consistent with other studies. Although operative vaginal delivery may be performed, as infrequently as in 1.5% of deliveries in some countries, it may be as high as 15% in other countries. For example, in the United Kingdom, the rates of instrumental vaginal delivery range between 10% and 15%; these rates have remained fairly constant. Of the total 242 OVDs, forceps and vacuum deliveries account for 8.2% and 2.1% of all the deliveries during the study period respectively with ratio of 4:1, but according to study done at Tikur Anbessa Hospital the ratio is 2:1 and it is not in line with the currently increasing proportion of vacuum deliveries which is 1:4. The higher difference in the proportion of forceps to vacuum deliveries from other studies can be due to the commonest indication being NRFHRP which needs faster delivery and the inconsistent supply of functioning vacuum extraction devices in the study area [1, 2, 5, 11].

Fetal distress (NRFHRP) was the commonest indication (56.2%) for OVD among the 242 cases followed by prolonged SSOL (24.0%) and those used to cut short SSOL (19.4%) and the finding is consistent with other studies. For example, a five-year retrospective study done on trends of instrumental deliveries at a tertiary care teaching hospital in Puducherry, India, shows among the study participants the indications were nonreassuring fetal heart (45.3%), prolonged second stage of labor (33.9%), and maternal indication to shorten second stage of labor (13.1%). The study done at the Tikur Anbessa Hospital also shows that the most common indication for OVD is fetal distress (45.3%) [2, 8].

According to our study the overall rate of complication is 17.3% (maternal=4.1% and neonatal=13.2%). The commonest maternal complication was postpartum hemorrhage (3.3%) and this can be explained by genital tract laceration which account for 62.5% of the PPH and prolonged labor also contributes PPH secondary to uterine atony. But the finding of rate of PPH among operative vaginal deliveries is much lower than study done at Aminu Kano Teaching Hospital, Kano, Nigeria, which is 9.5% and this may be due to lack of practice in documenting estimated blood loss and determining postoperative hematocrit after at least suspected PPH cases [2, 10].

Vacuum deliveries are associated with significant fetal morbidity and among vacuum deliveries fetal morbidity is 32% and 20% were complicated with subgaleal hemorrhage. The rates of severe birth asphyxia and ENND were 4.9% and 3.7%, respectively. This is compared to the findings of various studies; for example, according to study carried out at the Aminu Kano Teaching Hospital, Kano, Nigeria, the rate of asphyxia and ENND is 4.8% and 3.8%, respectively. But, this may not be truly attributable to the procedure as the asphyxia may be the outcome of the events of labor that indicated the intervention than the operative vaginal procedure itself [3, 10, 12, 13].

Among the 242 OVDs, neonates with low APGAR scores (4-6) at first and fifth minutes were 95(39.3%) and 16(6.6%), respectively, and those with very low APGAR score (0-3) at first and fifth minutes were 10(4.1%) and 2(0.8%), respectively. Compared to other studies the rate of low APGAR score (<7) is higher and this can be explained by the fact that fetal distress was the commonest indication for OVD according to our study and also the commonest cause of low APGAR scores at the 1^st^ and fifth minute among indications of operative vaginal deliveries [2, 14–16]. Our study and most other studies showed that there is significant association between type of instrument used for OVD and neonatal outcome, 80% of mothers who gave birth by vacuum are less likely to have favorable neonatal outcome than those with forceps deliveries. According to one of the studies, cephalhematoma, in particular, is more common after vacuum-assisted extraction than forceps delivery (approximately 15 versus 2 percent). The risks of fetal injury are generally instrument specific, with vacuum deliveries accounting for statistically significantly higher rates of cephalhematoma, and subgaleal and retinal hemorrhages, and forceps deliveries accounting for a no significantly higher rate of scalp/facial injuries [11, 17–20].

Meconium stained amniotic fluid had shown association with neonatal outcome, 90% of mothers with grade 3 MSAF are less likely to have favorable neonatal outcome than those with clear amniotic fluid. Studies showed that the association can be explained by passage of meconium secondary to already existing intrauterine fetal compromise or asphyxia [11, 21–23]. Neonatal birth weight had shown strong association with maternal outcome, 99.6% of those mothers who gave birth to neonate with birth weight >4000grams are less likely to have favorable maternal outcome when compared to those with normal birth weight. This finding as it is proven on different literatures, macrosomia attributes for PPH secondary to both uterine atony and perineal lacerations [4, 11, 24].

## 5. Conclusion and Recommendation

### 5.1. Conclusion

The prevalence of OVD among the 2348 laboring mothers who gave birth at JUMC during the study period is 10.3%. The commonest indication for OVD is NRFHRP (56.2%) followed by prolonged SSOL (24.0%) and shortening SSOL (19.8%). Among mothers who gave birth by OVD, 86.8% had favorable neonatal outcome. Near to all (95.9%) of mothers who gave birth by OVD had favorable maternal outcome. Type of instrument used for OVD and presence of MSAF are factors affecting neonatal outcome. Neonatal birth weight is significant factor affecting maternal outcome.

### 5.2. Recommendations

Nearby health facilities should be equipped with and use instruments needed for OVD as majority (59.5%) of mothers were referred from other facilities, application of OVD does not need referral to tertiary hospital. Although the ANC coverage among mothers with OVD is higher (96.3% had at least one ANC visit and 48.8% had four and above ANC visit) than the national and Oromia figure, mothers should be encouraged to have the recommended number of ANC follow-up and further study is needed to know and address the reason why mothers are not having the ANC visit as per the recommendation. The ratio of forceps to vacuum delivery according to this study is 4:1 but currently vacuum has worldwide acceptance because of technical simplicity to apply and relatively less maternal trauma, so using vacuum for OVD should be encouraged. Documenting estimated blood loss and determining postoperative hematocrit after at least suspected PPH cases should be practiced by health professionals attending OVDs, as none of patients' charts contain adequate data about the circumstance of delivery and that can be evidenced by relatively lower rate of PPH. The commonest maternal complication was postpartum hemorrhage (3.3%). The PPH cases were higher among mothers who gave birth to neonates with birth weight >4000grams and the study had shown the association. Thus, there is need to anticipate postpartum hemorrhage in operative vaginal deliveries special in case macrosomia suspected.

## Figures and Tables

**Table 1:** Frequency distribution of sociodemographic characteristics of mothers who gave birth by OVD in JUMC December 1, 2016–May 30, 2017.

Variable	Variable category	Frequency	Percent
Age	15-19	27	11.2
	20-24	92	38.0
	25-29	79	32.6
	30-34	31	12.8
	35-39	13	5.4

Place of residency	Jimma Town	98	40.5
	Outside Jimma town	144	59.5

Ethnicity	Oromo	201	83.1
	Amhara	13	5.4
	Tgrie	3	1.2
	Gurage	7	2.9
	Dawuro	12	5.0
	Other	6	2.5

Religion	Muslim	161	66.5
	Orthodox	55	22.7
	Protestant	24	9.9
	Other	2	.8

Occupation	Housewife	101	41.7
	civil servant	39	16.1
	Farmer	82	33.9
	Merchant	11	4.5
	Other	9	3.7

Educational status	Illiterate	77	31.8
	read and write only	4	1.7
	grade 1-8	81	33.5
	grade 9-10	25	10.3
	grade11-12	31	12.8
	>12	24	9.9

Income of the family per month	500 ETB and below	10	4.1
	501-1742 ETB	128	52.9
	above 1742	104	43.0

Marital status	Married	237	97.9
	Single	3	1.2
	Divorced	1	.4
	Widowed	1	.4

**Table 2:** Frequency distribution of obstetric related variables among mothers who gave birth by OVD in JUMC December 1, 2016–May 30, 2017.

Obstetric related	Variable category	Frequency	Percent
Parity	I	168	69.4
	II-IV	53	21.9
	≥V	21	8.7

GA	pre-term	14	5.8
	Term	213	88.0
	post term	15	6.2

ANC follow up	yes	233	96.3
	no	9	3.7

Number of ANC follow up	one visit	4	1.7
	two to three visit	111	45.9
	four and above visit	118	48.8
	No visit	9	3.7

Type of OVD used	vacuum	50	20.7
	forceps	192	79.3

Type of OVD applied	low	132	54.5
	outlet	110	45.5

Time of application	on arrival	69	28.5
	followed	173	71.5

Status of liquor	clear	172	71.1
	G1MSAF	19	7.9
	G2MSAF	17	7.0
	G3MSAF	34	14.0

Weight of the newborn in grams	1500-2499	14	5.8
	2500-3999	221	91.3
	>/=4000	7	2.9

**Table 3:** Distribution of neonatal outcome among different maternal sociodemographic characteristic categories who gave birth by OVD in JUMC December 1, 2016–May 30, 2017.

**Variable**	**Variable category**	**Neonatal outcome**		**Total**
		**Favorable outcome N (**%**)**	**Unfavorable outcome N (**%**)**	
Age	15-19	24(88.9)	3(11.1)	27(100.0)
	20-24	81(88.0)	11(12.0)	92(100.0)
	25-29	71(89.9)	8(10.1)	79(100.0)
	30-34	25(80.6)	6(19.4)	31(100.0)
	35-39	9(69.2)	4(30.8)	13(100.0)

Place of the residency	Jimma Town	90(91.8)	8(8.2)	98(100.0)
	Outside Jimma town	120(83.3)	24(16.7)	144(100.0)

Occupation	Housewife	95(94.1)	6(5.9)	101(100.0)
	civil servant	33(84.6)	6(15.4)	39(100.0)
	Farmer	64(78.0)	18(22.0)	82(100.0)
	Merchant	9(81.8)	2(18.2)	11(100.0)
	Other	9(100.0)	0(0.0)	9(100.0)

Educational status	Illiterate	61(79.2)	16(20.8)	77(100.0)
	read and write only	4(100.0)	0(0.0)	4(100.0)
	grade 1-8	75(92.6)	6(7.4)	81(100.0)
	grade 9-10	21(84.0)	4(16.0)	25(100.0)
	grade11-12	27(87.1)	4(12.9)	31(100.0)
	>12	22(91.7)	2(8.3)	24(100.0)

Income of the family per month	500 ETB and below	9(90.0)	1(10.0)	10(100.0)
	501-1742 ETB	103(80.5)	25(19.5)	128(100.0)
	above 1742ETB	98(94.2)	6(5.8)	104(100.0)

Marital status	Married	205(86.5)	32(13.5)	237(100.0)
	Others	5(100.0)	0(0.0)	5(100.0)

**Table 4:** Distribution of neonatal outcome among mothers who gave birth by OVD in JUMC December 1, 2016–May 30, 2017.

variable	Variable category	Neonatal outcome		Total
		Favorable outcome N (%)	Unfavorable outcome N (%)	
Parity	I	146(86.9)	22(13.1)	168(100.0)
	II-IV	51(96.2)	2(3.8)	53(100.0)
	≥V	13(61.9)	8(38.1)	21(100.0)

GA in weeks	pre-term	12(85.7)	2(14.3)	14(100.0)
	Term	186(87.3)	27(12.7)	213(100.0)
	post term	12(80.0)	3(20)	15(100.0)

ANC follow up	yes	205(88.0)	28(12.0)	233(100.0)
	No	5(55.6)	4(44.4)	9(100.0)

Number of ANC follow up	one visit	3(75.0)	1(25.0)	4(100.0)
	two to three visit	94(84.7)	17(15.3)	111(100.0)
	four and above visit	108(91.5)	10(8.5)	118(100.0)
	no visit	5(55.6)	4(44.4)	8(100.0)

Indication for OVD	NRFHRP	115(84.6)	21(15.4)	136(100.0)
	shortening SSOL	44(91.7)	4(8.3)	48(100.0)
	prolonged SSOL	51(87.9)	7(12.1)	58(100.0)

Type of OVD used	vacuum	34(68.0)	16(32.0)	50(100.0)
	forceps	176(91.7)	16(8.3)	192(100.0)

Type of OVD applied	Low	110(83.3)	22(16.7)	132(100.0)
	outlet	100(90.9)	10(9.1)	110(100.0)

Time of application	on arrival	52(75.4)	17(24.6)	69(100.0)
	followed	158(91.7)	15(8.3)	173(100.0)

Status of liquor	clear	163(94.8)	9(5.2)	172(100.0)
	G1MSAF	17(89.5)	2(10.5)	19(100.0)
	G2MSAF	13(76.5)	4(23.5)	17(100.0)
	G3MSAF	17(50.0)	17(50.0)	34(100.0)

weight of the newborn in grams	1500-2499	13(92.9)	1(7.1)	14(100.0)
	2500-3999	192(86.9)	29(13.1)	221(100.0)
	>/=4000	5(71.4)	2(28.6)	7(100.0)

**Table 5:** Distribution of maternal outcome cross tabulated with sociodemographic characteristics among mothers who gave birth by OVD in JUMC December 1, 2016–May 30, 2017.

Variables with category		Maternal outcome		Total
		Favorable outcome N (%)	Unfavorable outcome N (%)	
Age	15-19	26(96.3)	1(3.7)	27(100.0)
	20-24	90(97.8)	2(2.2)	92(100.0)
	25-29	74(93.7)	5(6.3)	79(100.0)
	30-34	30(96.8)	1(3.2)	31(100.0)
	35-39	12(92.3)	1(7.7)	13(100.0)

Place of the residency	Jimma Town	98(100.0)	0(0.0)	98(100.0)
	Outside Jimma town	134(93.1)	10(6.9)	144(100.0)

Occupation	Housewife	97(96.0)	4(4.0)	101(100.0)
	civil servant	39(100.0)	0(0.0)	39(100.0)
	Farmer	76(92.7)	6(7.3)	82(100.0)
	Merchant	11(100.0)	0(0.0)	11(100.0)
	Other	9(100.0)	0(0.0)	9(100.0)

Educational status	Illiterate	71(92.2)	6(7.8)	77(100.0)
	read and write only	4(100.0)	0(0.0)	4(100.0)
	grade 1-8	78(96.3)	3(3.7)	81(100.0)
	grade 9-10	24(96.0)	1(4.0)	25(100.0)
	grade11-12	31(100.0)	0(0.0)	31(100.0)
	>12	24(100.0)	0(0.0)	24(100.0)

Income of the family per month	500 ETB and below	8(80.0)	2(20.0)	10(100.0)
	501-1742 ETB	122(95.3)	6(4.7)	128(100.0)
	above 1742	102(98.1)	2(1.9)	104(100.0)

Marital status	Married	227(95.8)	10(4.2)	237(100.0)
	Others	5(100.0)	0(0.0)	5(100.0)

**Table 6:** Distribution of maternal outcome cross tabulated with maternal obstetric related variables among mothers who gave birth by OVD in JUMC December 1, 2016–May 30, 2017.

Obstetric related factors		Maternal outcome		Total
		Favorable outcome N (%)	Unfavorable outcome N (%)	
Parity	I	160(95.2)	8(4.8)	168(100.0)
	II-IV	52(98.1)	1(1.9)	53(100.0)
	≥V	20(95.2)	1(4.8)	21(100.0)

ANC follow up	Yes	223(95.7)	10(4.3)	233(100.0)
	No	9(100.0)	0(0.0)	9(100.0)

Indication for OVD	NRFHRP	132(97.1)	4(2.9)	136(100.0)
	shortening SSOL	43(89.6)	5(10.4)	48(100.0)
	prolonged SSOL	57(98.3)	1(1.7)	58(100.0)

Type of OVD used	Vacuum	48(96.0)	2(4.0)	50(100.0)
	Forceps	184(95.8)	8(4.2)	192(100.0)

Type of OVD applied	Low	123(93.2)	9(6.8)	132(100.0)
	Outlet	109(99.1)	1(.9)	110(100.0)

Time of application	on arrival	67(97.1)	2(2.9)	69(100.0)
	Followed	165(95.4)	8(4.6)	173(100.0)

weight of the newborn in grams	1500-2499	12(85.7)	2(14.3)	14(100.0)
	2500-3999	213((96.4)	8(3.6)	221(100.0)
	>/=4000	7(100.0)	0(0.0)	7(100.0)

**Table 7:** Factors affecting neonatal outcome using multivariate logistic regression among mothers who gave birth by OVD in JUMC December 1, 2016–May 30, 2017.

Variables with category		Neonatal outcome		Crude OR(95% CI)	AOR(95%CI)	P value
		Fav	Unfav			
Place of the residency	Jimma Town	90	8	2.250(.966,5.241)	.653(.176,2.429)	0.525
	Outside Jimma town	120	24	1	1	

Occupation	Housewife	95	6	1	1	
	civil servant	33	6	.347(.105, 1.152)	0.282(.062,1.278)	0.101
	Farmer	64	18	.225(.085, .596)	0.299(.061,1.462)	0.136
	Merchant	9	2	.284(.050, 1.620)	0.221(.029,1.692)	0.146
	Other	9	0	1.020(.000,2.310)	1.110(.000,3.23)	0.999

Income of the family per month	500 ETB and below	9	1	1.000(.118, 8.487)	4.650(.248,87.109	0.304
	501-1742 ETB	103	25	1	1	
	above 1742	98	6	.490(229, 1.051)	1.551(.325, 7.397)	0.582

Parity	I	146	22	1	1	
	II-IV	51	2	3.842(.873,16.917)	2.719(.525,14.070)	0.233
	≥V	13	8	.245(.091, .658)	0.294(.072, 1.197)	0.088

Indication for OVD	NRFHRP	115	21	1	1	
	shortening SSOL	44	4	2.009(.653, 6.183)	0.698(.155, 3.146)	0.640
	prolonged SSOL	51	7	1.330(.532, 3.328)	0.906(.198, 4.143)	0.899

Type of OVD used*∗*	Vacuum	34	16	.193(.088, .423)	0.228(0.078, 0.671)	0.007
	Forceps	176	16	1	1	

Type of OVD applied	Low	110	22	1	1	
	Outlet	100	10	2.000(.903, 4.429)	1.604(.577, 4.458)	0.365

Time of application	on arrival	52	17	.290(.136, .622)	0.436(.118, 1.615)	0.214
	Followed	158	15	1	1	

Status of liquor*∗*	Clear	163	9	1	1	
	G1MSAF	17	2	.469(.094, 2.352)	0.574(.081, 4.088)	0.579
	G2MSAF	13	4	.179(.049, .663)	0.163(.031, .858)	0.032
	G3MSAF	17	17	.055(.021, .143)	0.088(.024, .327)	0.000

*∗* shows significant predictor variable with p<0.05.

**Table 8:** Factors affecting maternal outcome using multivariate logistic regression among mothers who gave birth by OVD in JUMC December 1, 2016–May 30, 2017.

Variables with category		Maternal outcome		Crude OR(95% CI)	AOR(95%CI)	P value
		Fav	Unfav			
Income of the family per month	500 ETB and below	8	2	0.058(0.007,0.467)	0.004(.000, 0.133)	0.057
	501-1742 ETB	122	6	1	1	
	above 1742ETB	102	2	0.208(0.041,1.053)	0.060(0.004, 0.961)	0.073

Indication for OVD	NRFHRP	132	4	1	1	
	shortening SSOL	43	5	0.261(0.067, 1.014)	0.166(0.026,1.067)	0.058
	prolonged SSOL	57	1	1.727(0.189,15.796)	1.632(.146,18.298)	0.691

Type of OVD applied	Low	123	9	1	1	
	Outlet	109	1	7.976(0.994, 63.969)	8.262(0.670,101.942)	0.100

weight of the newborn in grams*∗*	1500-2499	12	2	0.167(0.031, 0.919)	0.355(0.050, 2.546)	0.303
	2500-3999	213	8	1	1	
	>/=4000	7	0	0.070(0.011, 0.435)	0.007(0.000, 0.151)	0.002

*∗* shows significant predictor variable with p<0.05; Fav: favorable; Unfav: unfavorable.

## Data Availability

The data used to support the findings of this study are included within the article.

## Abbreviations

ANC: Antenatal care
ARM: Artificial rupture of membrane
FHR: Fetal heart rate
IUFD: Intrauterine fetal death
ENND: Early neonatal death
EFW: Estimated fetal weight
GA: Gestational age
HCT: Hematocrit
HTN: Hypertension
JUMC: Jimma University Medical Center
MSAF: Meconium stained amniotic fluid
NICU: Neonatal intensive care unit
ObGy: Obstetrics and Gynecology
OL: Obstructed labor
OVD: Operative vaginal delivery
PE: Preeclampsia
PPH: Postpartum hemorrhage
PNA: Perinatal asphyxia
ROM: Rupture of membrane
SSOL: Second stage of labor
SVD: Spontaneous vaginal delivery
SGH: Subgaleal hemorrhage
WHO: World Health Organization.
